# *C9ORF135* encodes a membrane protein whose expression is related to pluripotency in human embryonic stem cells

**DOI:** 10.1038/srep45311

**Published:** 2017-03-27

**Authors:** Shixin Zhou, Yinan Liu, Yumin Ma, Xiaoyan Zhang, Yang Li, Jinhua Wen

**Affiliations:** 1 Department of Cell Biology and Stem Cell Research Center, School of Basic Medical Sciences, Peking University Health Science Center, Beijing 100191, China; 2 Department of Endocrinology and Metabolism, Peking University People’s Hospital, Peking University Diabetes Center, Beijing 100044, China

## Abstract

Human embryonic stem cells (hESCs) are a unique population of cells defined by their capacity for self-renewal and pluripotency. Here, we identified a previously uncharacterized gene in hESCs, *C9ORF135*, which is sharply downregulated during gastrulation and gametogenesis, along with the pluripotency factors OCT4, SOX2, and NANOG. Human ESCs express two *C9ORF135* isoforms, the longer of which encodes a membrane-associated protein, as determined by immunostaining and western blotting of fractionated cell lysates. Moreover, the results of chromatin immunoprecipitation (ChIP), mass spectrometry (MS), and co-immunoprecipitation (co-IP) analyses demonstrated that *C9ORF135* expression is regulated by OCT4 and SOX2 and that *C9ORF135* interacts with non-muscle myosin IIA and myosin IIB. Collectively, these data indicated that *C9ORF135* encodes a membrane-associated protein that may serve as a surface marker for undifferentiated hESCs.

Human embryonic stem cells (hESCs) are pluripotent cells typically derived from the inner cell mass of blastocyst-stage embryos^1^. These cells have the capacity to maintain self-renewal but they can also differentiate into ectoderm^2^,^3^,^4^, mesoderm^5^,^6^, and endoderm^7^,^8^, in addition to primordial germ cells (PGCs)^9^ and embryonic germ cells^9^,^10^. Understanding the transcriptional cues that direct hESC differentiation is essential for future clinical applications^11^,^12^.

Subtractive gene expression profiling comparing differentiated and undifferentiated cells has identified several potential regulators of the differentiation process, such as OCT4, NANOG, and SOX2, which form a transcriptional regulatory circuit necessary to maintain hESCs pluripotency^13^. In addition, epigenetic changes have been proposed to direct hESC differentiation^14^,^15^,^16^. However, the specific proteins that support the specific morphology of hESCs have not yet been identified. Previous work has sought to identify hESC surface marker proteins to facilitate the identification of these cells^17^; the identified markers include E-cadherin, epithelial cell adhesion molecule (EpCAM), and P-cadherin^18^. Most of these surface markers are also expressed in epithelial cells. Therefore, examination of the transcriptomes of hESCs and their differentiated counterparts has been regarded as an alternative method for screening specific hESC surface markers.

In this study, we performed a large-scale transcriptional analysis with gene expression profiles of undifferentiated hESCs, embryoid bodies, and progenies of cell lineages obtained from the GEO^11^ and ArrayExpress^12^ databases, focusing on uncharacterized genes that either contain putative transmembrane domains or are downregulated during hESC differentiation. In this analysis, we identified a previously uncharacterized gene, *C9ORF135*, which has been found in seven independent studies to be highly expressed in hESCs but to decrease rapidly in expression after cell differentiation. In the Swiss-Prot annotation database^19^, sequence analysis suggests that *C9ORF135* encodes a protein with a single putative transmembrane domain (http://www.uniprot.org/uniprot/Q5VTT2).

## Results

### Candidate indicators of human embryonic stem cell differentiation identified by gene expression profiling

**Figure 1:**
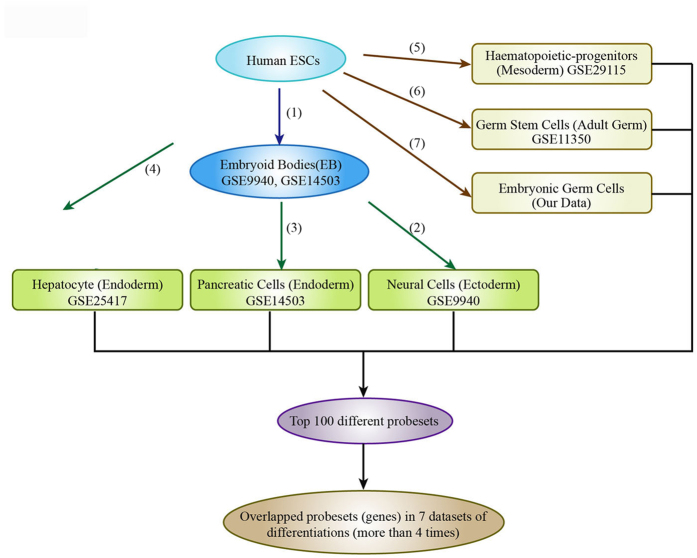
Schematic of gene expression profiling method for human embryonic stem cells (hESCs) and their differentiated derivatives. The hESCs were differentiated into three germ layers or germ cells directly or indirectly via EB stage. GEO dataset identifiers are labeled in the box. The numbers beside each line indicates the mean number of independent experiments for the sample set in our meta-analysis.

We downloaded six microarray datasets examining gene expression in hESCs, cells from the three differentiated germ layers, and PGC from the GEO database. Our data for hESCs (LiY) and embryonic germ cells^10^ were generated with Affymetrix U133 Plus 2 microarrays. These microarray data for differentiated hESCs with the same platform (U133 Plus 2) were collected and further analyzed to determine fold changes between hESCs and differentiated cells by expression profiling. The top 100 most differentially expressed genes were identified from each study, and those found in 4 or more studies were recorded for further analysis (Fig. 1; Table 1). Notably, we found that *C9ORF135* was sharply downregulated during differentiation in all seven studies, along with other known pluripotency genes (*NANOG, SOX2*), HLA (*HLA-DPB2*), and adhesion molecules (*CD24, CDH1*).

### Two *C9ORF135* isoforms are expressed in hESCs

**Figure 2:**
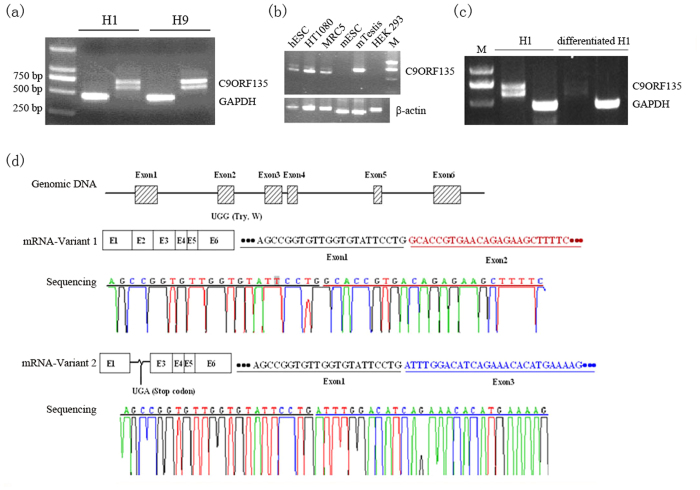
*C9ORF135* gene structure and isoforms. (**a**) The two *C9ORF135* isoforms in H1 and H9 hESCs*. GAPDH* served as an internal control. (**b**) *C9ORF135* expression in various cell lines and tissues. (**c**) RT-qPCR analysis of C9ORF135 expression during H1 hESC differentiation. (**d**) *C9ORF135* gene structure and its two isoforms. *C9ORF135* consists of six exons. Isoform 1 includes exons 1 and 2, whereas isoform 2 includes exon 1 and 3. The junction of exons 1 and 3 form a “TGA” stop codon.

To confirm our microarray findings, we designed primers to the *C9ORF135* coding sequence (NM_001010940) and found two distinct transcripts expressed in the hESC lines H9 and H1 (Fig. 2a). Further analysis showed that *C9ORF135* was also expressed in human MRC5 normal lung and HT1080 fibrosarcoma cells, but not in HEK293A cells. Interestingly, 1700028P14Rik, the mouse homolog of *C9ORF135*, displayed no or low expression in mouse ESCs but was highly expressed in the testis (Fig. 2b). Nevertheless, in hESCs, the expression of both transcripts was dramatically decreased when cells were subjected to retinoic acid (RA)-induced neural differentiation (Fig. 2c). The human *C9ORF135* loci structure is shown in Fig. 2d. Isoform 1 (full-length) has 6 exons and is 690 nt in size, whereas isoform 2 has a stop codon (TGA) in the junction of exons 1 and 3, owing to the lack of exon 2. Isoform 2 of C9ORF135 encodes a short peptide of only 50 amino acid residues, because of a stop codon at nt 151–153 (Fig. 2d). Therefore, we chose to explore the functions of isoform 1 and its encoded protein (Swiss-Prot ID: Q5VTT2) in hESCs.

### C9ORF135 protein localizes to the cytoplasm and plasma membrane in hESCs

**Figure 3:**
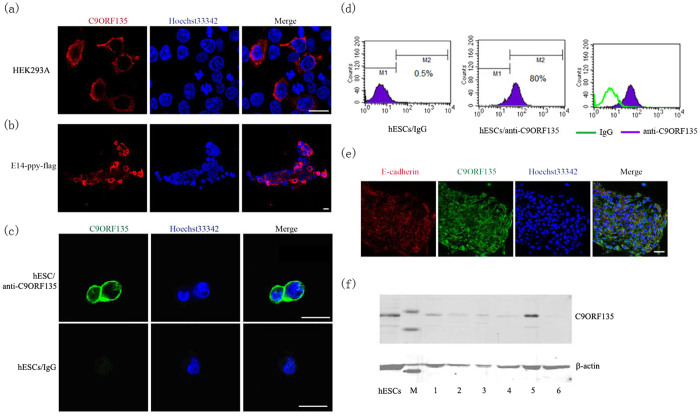
Subcellular localization of C9ORF135 protein. (**a**) Ectopic C9ORF135 expression in HEK293 cells. C9ORF135 (left, red); DAPI (middle, blue); Merge (right). Scale bars, 25 μm. (**b**) Localization of exogenous C9ORF135 in mouse E14 cells. Scale bars, 25 μm. (**c**) Localization of endogenous C9ORF135 protein in hESCs. (**d**) Flow cytometry analysis for hESCs stained with C9ORF135 antibody. (**e**) C9ORF135 and E-cadherin immunofluorescence in hESCs. Scale bars, 25 μm. A typical result from triplicate experiments is shown. (**f**) Subcellular fractionation and western blot analysis of hESCs. Lanes from left to right: hESC, total lysate; M, protein marker; 1, plasma membrane; 2, nucleus; 3, cytoskeleton (short lysis); 4, cytoskeleton (prolonged lysis); 5, cytoplasm; 6, HEK293A negative control cells. A typical result from triplicate experiments is shown.

C9ORF135 is not expressed in HEK293A cells. Hence, we exogenously expressed full-length *C9ORF135* (isoform 1) with an N-terminal FLAG tag and confirmed its localization in the plasma membrane and cytoplasm by anti-FLAG immunofluorescence (Fig. 3a). This staining pattern was also observed in mouse E14 ESCs with exogenous expression of 1700028P14Rik (Fig. 3b). Then, we tested the specificity of the C9ORF135 polyclonal antibody by western blotting analysis in HEK293A cells after overexpression of C9ORF135 (Fig. S1). A unique band was detected by the C9ORF135 antibody (Rabbit) in a similar position to that identified by the anti-FLAG monoclonal antibody (mouse). This result indicated the specificity of the C9ORF135 antibody and its ability to recognize the target protein. This observation was also consistent with the RT-PCR analysis showing that C9ORF135 was not expressed in HEK293A cells (Fig. 2b). Then, the H1 hESCs were digested with collagenase IV and stained with anti-C9ORF135 antibody. Further immunofluorescence showed that the endogenously expressed protein also displayed a similar localization pattern (Fig. 3c). These stained cells were analyzed by flow cytometry. The results showed there were approximately 79.5% cells that were C9ORF135 positive among the H1 hESCs (Fig. 3d). Double staining of C9ORF135 and E-cadherin in H1 hESCs indicated that C9ORF135 colocalized with E-cadherin at the plasma membrane (Fig. 3e). We also performed subcellular fractionation of H1 hESCs to isolate proteins in the various cell compartments, namely, the cytosol, membrane and organelles, nucleus, and cytoskeleton, and examined C9ORF135 localization by western blotting. These results also indicated that the C9ORF135 protein localized to the cytoplasm and plasma membrane of hESCs (Fig. 3f).

### Identification of C9ORF135 interactors

**Figure 4:**
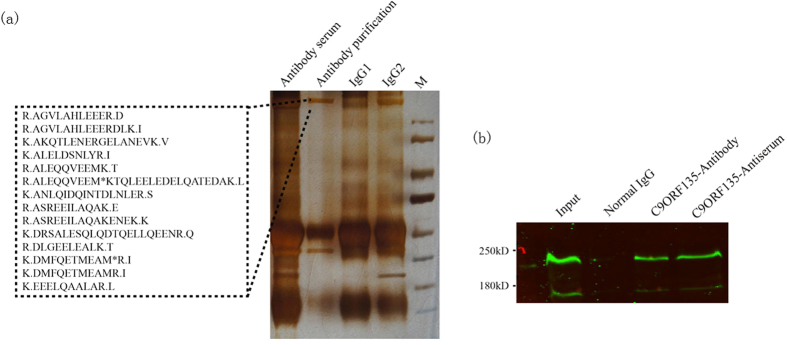
Identification of C9ORF135-interacting proteins. (**a**) Silver staining of C9ORF135 immunoprecipitated proteins separated by SDS-PAGE. Lanes from left to right: anti-C9ORF135, normal IgG (2 lanes), and protein marker. (**b**) C9ORF135 co-IP analysis in hESCs with myosin IIA antibody. Lanes from left to right: Input (10% lysates), normal IgG pull-down, and anti-C9ORF135 pull-down. A typical result from triplicate experiments is shown.

We sought to identify C9ORF135 binding proteins by mass spectrometry (MS) and co-immunoprecipitation (co-IP) analyses. First, C9ORF135 antibody, anti-serum, and control IgG pulldowns were analyzed by SDS-PAGE and silver staining. We then excised three bands present only in the antibody pulldown (Fig. 4a) and performed a molecular analysis by MS, which identified the bands as myosin-9 (MYH9, myosin-IIA) and myosin-10 (MYH10, myosin-IIB), belonging to a family of conventional non-muscle myosins involved in cytokinesis, cell motility and maintenance of cell shape^20^. Subsequent co-IP experiments confirmed myosin-IIA as a bona fide interactor of C9ORF135 in hESCs (Fig. 4b).

### Pluripotency factors OCT4 and SOX2 regulate C9ORF135 expression

**Figure 5:**
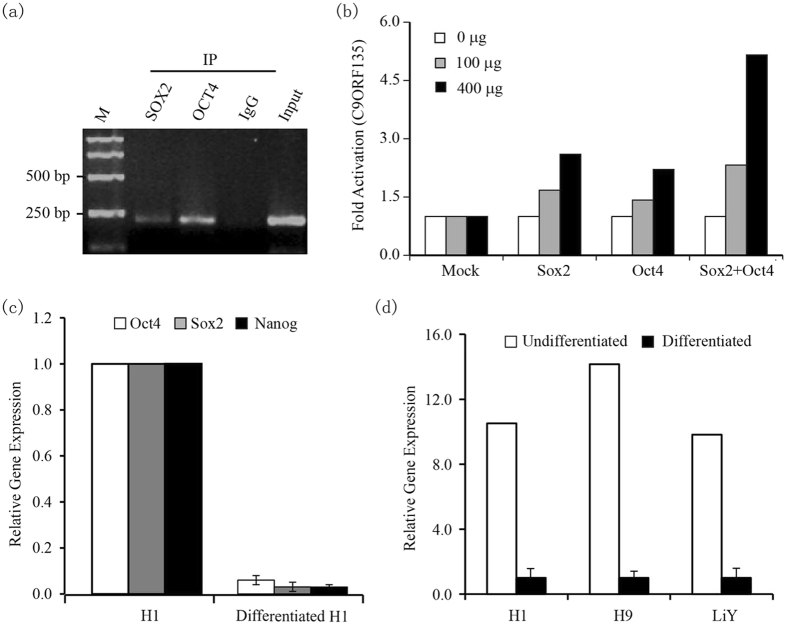
SOX2 and OCT4 synergistically regulate *C9ORF135* expression in hESCs. (**a**) Chromatin immunoprecipitation (ChIP) analysis was conducted using SOX2 and OCT4 antibodies and *C9ORF135*-specific primers. (**b**) *C9ORF135* promoter activity in the presence or absence of OCT4 and SOX2. (**c**) OCT4, NANOG, and SOX2 expression analysis in differentiated H1 hESCs. (**d**) C9ORF135 expression analysis in H1, H9, and LiY ESCs differentiated with retinoic acid (RA) for 7 days. The result shown is the average of triplicate experiments.

OCT4 and SOX2 are transcriptional regulators essential for maintaining hESC pluripotency. To determine whether C9ORF135 expression is regulated by OCT4 and SOX2, we performed chromatin immunoprecipitation (ChIP) in H1 hESCs and found that both SOX2 and OCT4 occupied the *C9ORF135* promoter in these cells (Fig. 5a). Additionally, we generated a luciferase reporter containing the *C9ORF135* proximal promoter to monitor transcriptional activity in the presence or absence of exogenous OCT4 and SOX2. Notably, both transcription factors induced luciferase activity in a synergistic and dose-dependent manner (Fig. 5b), thus indicating that SOX2 and OCT4 promote C9ORF135 expression by direct or indirect promoter binding. Subsequent analysis also showed that C9ORF135, as well as the core pluripotency factors OCT4, SOX2, and NANOG, were downregulated in H1, H9, and LiY ESCs treated with RA for seven days to induce differentiation (Figs. 5c,d). However, NANOG neither had transcriptional activity at the C9ORF135 promoter nor showed synergistic effects with OCT4 in the luciferase reporter system (Fig. S2). This finding further confirmed that C9ORF135 expression is regulated by OCT4 and SOX2 in hESCs.

### Decreased expression of *C9ORF135* in early stages of hESC differentiation

**Figure 6:**
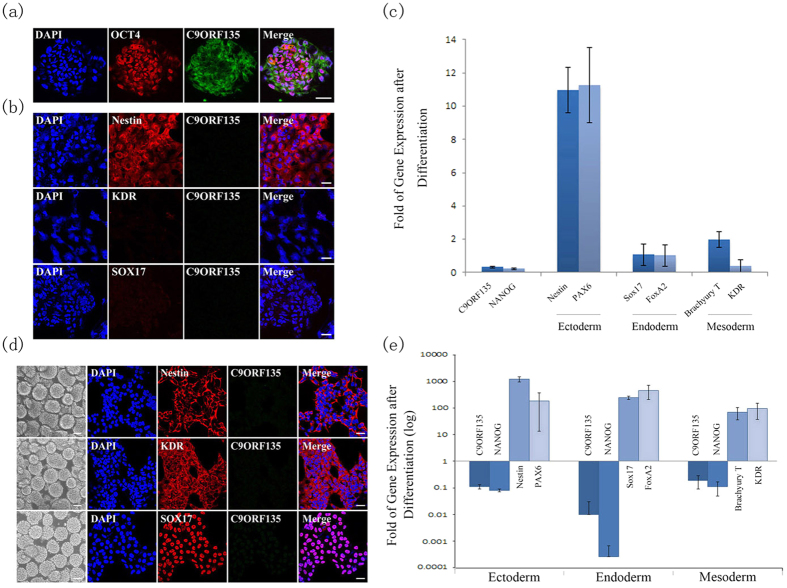
*C9ORF135* is expressed in undifferentiated hESCs and is downregulated during hESC differentiation (**a**) *C9ORF135* expression in undifferentiated ESCs. OCT4 (red), C9ORF135 (green) and merged images in undifferentiated ESCs. (**b**) C9ORF135 is downregulated during spontaneous differentiation of hESCs. EBs derived from H1 hESCs were digested with Accutase into single cells, seeded on Matrigel-coated glass-bottom dishes and subjected to immunofluorescence analysis. The lineage-specific markers were stained in red and C9ORF135 in green. Nuclei (DAPI, blue). Scale bars, 50 μm. A typical result from triplicate experiments is shown. (**c**) C9ORF135 mRNA level is downregulated in spontaneous differentiation of hESCs. Nestin and Pax6 are ectoderm markers. KDR and Brachyury T are mesoderm markers. Sox17 and FoxA2 are endoderm markers. Mean values + SD are shown in the figure. (**d**) C9ORF135 is downregulated during differentiation into three germ layers. EBs derived from H1 hESCs were cultivated in the designated induction medium (phase-contrast images, 4d) in left panels. EBs were digested with Accutase, and the resultant single cells were seeded on Matrigel-coated glass-bottom dishes. Immunofluorescence assay was performed for identifying the pluripotent or specific lineage markers. The lineage-specific markers are labeled in red, and C9ORF135 is shown in green. Nuclei were stained with DAPI (blue). Scale bars, 50 μm. A typical result from triplicate experiments is shown. (**e**) The C9ORF135 mRNA level is dramatically downregulated during differentiation into three germ layers. The mRNA levels of lineage-specific markers are substantially increased during the differentiation to ectoderm, endoderm or mesoderm cells. The vertical coordinate is a logarithmic scale. Mean values + SD are shown in the figure.

We repeated the immunostaining experiment in H1 hESCs with antibodies to C9ORF135 and OCT4 (pluripotent marker). The results again showed that C9ORF135 and OCT4 were expressed in hESCs (Fig. 6a). In addition, we induced spontaneous differentiation in H1 hESCs by withdrawing bFGF and TGFβ from the E6 culture medium. The H1 cells formed embryoid bodies (EBs), which were cultivated for 4 days under the same conditions. We collected cells from the ESCs and EBs to compare their mRNA and protein levels of the pluripotency marker NANOG and three germ layer markers, as well as C9ORF135. The immunostaining analyses showed that the ectoderm marker Nestin was strongly positive in the EBs formed from spontaneous differentiation, whereas C9ORF135 was significantly downregulated. However, the EBs exhibited negative staining for the mesoderm marker KDR and the endoderm marker Sox17 (Fig. 6b). The results of RT-qPCR were similar to the results of immunostaining analyses. The expression of NANOG (pluripotency marker) decreased for EBs, similar to C9ORF135. Expression levels of Nestin and Pax6 (ectoderm markers) increased substantially for EBs. However, we observed only minor fluctuations at the mRNA level for endoderm (FoxA2 and Sox17) and mesoderm (Brachyury T and KDR) markers (Fig. 6c). Therefore, the results of immunostaining analyses and RT-qPCR indicated that hESCs were inclined toward the ectoderm lineage cells during the spontaneous differentiation.

Then, we established a 4-day designated protocol to induce the differentiation of EBs toward the three germ layers in order to observe the expression of C9ORF135 in the early stage of differentiation. Lineage-specific markers were used to identify EBs both at the mRNA and protein levels. The results of double immunostaining and RT-qPCR analyses indicated that the expression of C9ORF135 was significantly downregulated during differentiation. The lineage-specific markers Nestin (ectoderm), KDR (mesoderm) and Sox17 (endoderm) are upregulated substantially as shown by immunostaining analyses (Fig. 6d). Meanwhile, the mRNA levels of lineage-specific markers, Nestin and Pax6 for ectoderm, Sox17 and FoxA2 for endoderm, and Brachyury T and KDR for mesoderm, increased dramatically at the early differentiation stage of EBs, as demonstrated by RT-qPCR analysis (Fig. 6e). The expression levels of pluripotency marker NANOG and C9ORF135 decreased sharply during the designated differentiation process (Fig. 6e).

### Morphological alterations in differentiation after *C9ORF135* knockdown in hESCs

**Figure 7:**
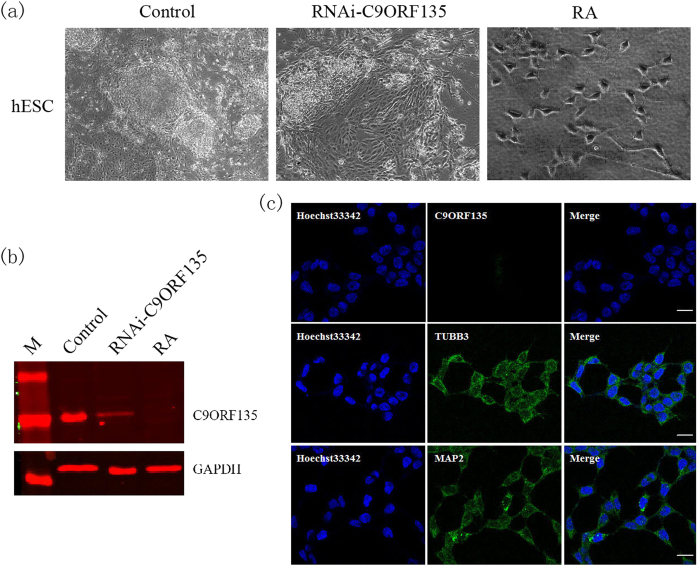
*C9ORF135* is downregulated in response to hESC differentiation. (**a**) Morphology and (**b**) western blot analysis of hESC clones after *C9ORF135* knockdown or treatment with RA. (**c**) Immunofluorescence analysis of C9ORF135 and neural cell markers TUBB3 and MAP2 in differentiated hESCs.

Our results indicated that the specific shRNA to C9ORF135 decreased C9ORF135 expression and altered the morphology of hESCs (Fig. 7a and b). However, the scrambled shRNA changed neither the C9ORF135 expression level nor the morphology of hESCs (Fig. S3).

RA induces neural differentiation in ESCs^21^. Accordingly, hESCs displayed morphological spreading when the cultures were either treated with RA for seven days or subjected to *C9ORF135* shRNA knockdown (Fig. 7a). Analysis of RA-differentiated cells also showed downregulation of the C9ORF135 protein level (Fig. 7b) as well as an increase in the neural cell markers TUBB3 and MAP2 (Fig. 7c).

## Discussion

Human ESCs have the capacity to differentiate into all three germ layers^5^,^8^,^22^, a process during which their gene expression profiles change dramatically. In this study, we obtained microarray data from six studies analyzing hESC differentiation. A meta-analysis of these studies, in addition to our own data, revealed that the previously uncharacterized factor *C9ORF135* was markedly downregulated in response to hESC differentiation. Further examination showed that *C9ORF135* is expressed in two isoforms in hESCs and that the larger full-length protein localizes to the plasma membrane and cytosol. Notably, C9ORF135 expression in hESCs is regulated by the core pluripotency transcription factors SOX2 and OCT4, all of which are downregulated during RA-induced neural differentiation.

Human ESCs form round colonies with well-defined edges that are particularly sensitive to dissociation into single-cell suspensions, whereas mouse embryonic stem cells (mESCs) form smaller, irregular clones that are more resistant to clone disruption^23^,^24^. These physiological differences indicate that species-specific molecules may exist in hESCs. C9ORF135 is expressed throughout the cytoplasm and membranes of hESCs, but its mouse homolog is not detected in mESCs. As expected, the hESC clone morphology was markedly changed after *C9ORF135* knockdown and during differentiation. Additionally, MS and co-IP analyses suggest that C9ORF135 may interact with non-muscle myosin-9 (also known as myosin-IIA), which generates contractile tension to drive epithelial morphogenesis and support cell integrity^20^,^25^.

In conclusion, our results showed that *C9ORF135* is a membrane-associated protein. The expression of *C9ORF135* is regulated by OCT4 and SOX2 and decreases dramatically during hESC differentiation, thus suggesting that this protein may be a key regulator of pluripotency and may be used as a potential surface marker of hESCs.

## Methods

### Gene expression profiling

Microarray datasets of hESCs and differentiated cells were generated on the GPL570 platform (Affymetrix HG-U133 Plus 2.0; Affymetrix, Santa Clara, CA, USA) or were downloaded from GEO^11^; our analyses included data from hESCs, embryoid body (EB) and neural progenitor cells (GSE9940, ectoderm), pancreatic cells (GSE14503, endoderm)^26^, hepatocytes (GSE25417, endoderm)^8^, hematopoietic progenitor cells (GSE29115, mesoderm)^6^, and male adult germ stem cells (GSE11350)^9^.

We also performed a microarray analysis on LiY hESCs and embryonic germ cells on the Affymetrix Human U133 Plus 2.0 platform. For this analysis, 10 μg of total RNA was used as input for labeled cRNA synthesis, per the manufacturer’s instructions (IVT: 16 h). The quality-checked cRNA samples were hybridized in biological replicates for 18 h. All the steps were performed according to the manufacturer’s instructions, and the data were analyzed in Affymetrix Expression Console Software 1.1 (Affymetrix, Santa Clara, USA). The gene expression fold-change was assessed to select the top 100 differentially expressed genes between hESCs and differentiated cells for each downloaded study and our primary data.

### Cell culture and hESC differentiation with retinoic acid

HEK293A and HEK293T cells were cultured in Dulbecco’s modified Eagle’s medium (DMEM, HyClone Laboratories, Logan, UT, USA) containing 10% fetal bovine serum (FBS, Gibco, Grand Island, NY, USA). The hESC lines H1 and H9 were obtained from WiCell Research Institute (Madison, WI, USA), and the hESC line named LiY was established in our lab^10^. These hESCs were maintained on a layer of MEF (mouse embryonic fibroblast) feeder cells with 80% DMEM/F12 (Invitrogen, Carlsbad, CA, USA), 20% Knockout Serum Replacement (KSR), 1 mM l-glutamine, 1% non-essential amino acids, 0.1 mM β-mercaptoethanol (all from Invitrogen), and 4 ng/mL basic fibroblast growth factor (bFGF, Millipore, Temecula, CA, USA). Cell cultures were passaged with collagenase IV (Invitrogen), and the medium was changed every two days.

To induce hESC differentiation, hESCs were dissociated with 0.5 mM EDTA and then replated onto a bacteriological dish at 5 × 10^4^ cells/mL in 10 mL minimum essential medium (MEM alpha) (Gibco) supplemented with 10% FBS, 3 mM sodium bicarbonate, and 0.1 mM 2-ME. On day 2, retinoic acid (RA, 500 nM; Sigma, St. Louis, MO, USA) was added to the cultures and incubated for 7 days^27^. Cells at 0 and 7 days were used as hESCs and differentiated cells, respectively.

### RT-qPCR

RNA was extracted using an RNeasy Plus Mini kit (Qiagen, Hilden, Germany), per the manufacturer’s instructions. RT-qPCR was performed with SYBR Premix (Takara, Tokyo, Japan) with an MX3000 P PCR machine (Stratagene, San Diego, USA). The following thermocycling procedure was used for all PCR experiments: 95 °C for 5 min; 40 cycles at 95 °C for 30 s, annealing temperature 58–60 °C for 30 s; and terminated with a final extension at 72 °C for 10 min. The primers were as follows: *C9ORF135* forward, 5′-TGGTGTATTCCTGGCACCGT-3′ and reverse, 5′-GGGATTCATCGGTTCCCAGT-3′; NANOG forward, 5′-TGGCGCGGTCTTGGCTCACT-3′ and reverse, 5′-AGGTGGCGGGCGCCTGTAG-3′; SOX2 forward, 5′-GCCGAGTGGAAACTTTTGTCG-3′ and reverse, 5′-GGCAGCGTGTACTTATCCTTCT-3′; OCT4 forward, 5′-GTACTCCTCGGTCCCTTTCC-3′ and reverse, 5′-CAAAAACCCTGGCACAAACT-3′^28^.

Other RT-qPCR primers used to probe hESC differentiation are listed in Supplementary Table S1.

### Cloning and transfection

To generate *C9ORF135* expression vectors, the full-length cDNA was cloned into pcDNA3.1 containing an N-terminal FLAG tag with terminal BamHI and EcoRI restriction enzyme sites. PCR primers for C9ORF135 amplification are as follows: forward, 5′-GATGGATCCATGGATAGCCTTGACAGATC-3′ and reverse, 5′-GCGGAATTCTTAAATTGGCACAATAGGCC-3′. The C9ORF135 mouse homolog, 1700028P14Rik, was isolated from the testes of C57BL/6 mice by RT-PCR. The resulting cDNA was cloned into the episomal PpyCAGIP vector and transfected into E14 mouse embryonic stem cells with Lipofectamine 2000 (Invitrogen).

The C9ORF135 knockdown vector was generated by cloning CoORF135 shRNA (5′-CAGAAACUCUAUCCCUUGATT-3′) into the HpaI and XhoI sites of the pLL3.7 lentivirus vector with a U6 promoter. Lentiviral infection was performed by cotransfection of 15 μg of pLL3.7-shRNA together with 5 μg each of pMDLg/pRRE, RSV/Rev, and VSV-G into HEK293T cells using Lipofectamine 2000 (Invitrogen). The medium was changed 6 h later, and cultures were incubated for another 48 h. Culture supernatants were then collected, filtered with a 0.45-μm filter (Millipore) and then added to hESC cultures in the presence of 10 ng/μL polybrene (Sigma).

### Antibodies, immunofluorescence and flow cytometry

N-terminal *C9ORF135* peptides were used to immunize two rabbits over the course of one month. The immunized sera were collected and purified with an affinity column (Epitomics Inc., Burlingame, CA, USA). The purified antibody was used for immunofluorescence analysis alongside antibodies against TUBB3 and MAP2 (Chemicon, Temecula, CA, USA), E-cadherin (BD Biosciences, San Jose, CA, USA), and anti-FLAG (Sigma). The antibodies to Nestin, Sox17, and KDR were purchased from Abcam (Cambridge, UK), and anti-OCT4 was obtained from Santa Cruz Biotechnology (Santa Cruz, CA, USA). Alexa Fluor-labeled secondary antibodies were obtained from Invitrogen.

The digested H1 hESCs were washed twice with PBS plus 0.5% FBS. Then, the cells were stained with primary antibody against C9ORF135 (1:200) and the corresponding secondary antibody (goat anti-rabbit IgG). Approximately 10,000 cells were acquired with a BD FACSCalibur machine (BD Biosciences). Data were analyzed with FACSCalibur tools.

### Chromatin immunoprecipitation (ChIP) and western blot analyses

For ChIP analysis, 10^7^ infected ES cells were collected and fixed with 4% polyformaldehyde. Cell lysates were prepared by incubating the cells in lysis buffer (50 mM Tris-HCl pH 8.0, 150 mM NaCl, 0.5% NP40) for 30 min at 4 °C, and samples were centrifuged at 14,000 *g* for 30 min at 4 °C. The fragmented chromatin by sonication was then precleared with protein A and G Sepharose beads before immunoprecipitation with SOX2 and OCT4 antibodies bound to Sepharose beads. The bound material was washed, eluted, and purified with a Qiagen PCR purification kit. The samples were then analyzed by PCR with 1 μL of purified DNA and amplified for 36 cycles with the following primers: forward, 5′-TTCTTGTGCCCACTCCACTCTA-3′ and reverse, 5′-ACGAGAGAACATCCCCTGAAAC-3′.

For western blotting, the cells were lysed with ice-cold lysis buffer supplemented with protease inhibitor cocktail (Roche, Basel, Switzerland)^29^. The protein (50–80 μg) was resolved by 10% SDS-PAGE and then transferred to 0.2-μm PVDF membranes. After being blocked in 5% skimmed milk for 1 h, the membranes were incubated at 4 °C overnight with primary antibodies against C9ORF135 (Epitomics Inc.), GAPDH (Abcam, Cambridge, UK), myosin IIA (Abcam), and β-actin (Sigma). The membranes were washed with PBST buffer three times. After incubation in fluorescence-labeled secondary antibodies (Rockland, Limerick, PA, USA) for 1 h at room temperature, the membranes were imaged with an Odyssey western blotting system (LI-COR Bio, Lincoln, NE, USA).

### Luciferase reporter assays

The *C9ORF135* promoter was PCR-amplified from H1 hESC genomic DNA, digested with KpnI and HindIII, and then ligated into the pGL3-Basic reporter vector (Promega). The primers for *C9ORF135* promoter amplification were as follows:

forward, 5′-GCAGGTACCAGAAGGGACTTGCGATTCTTG-3′; reverse, 5′-GCGAAGCTTAACCAGTGTTGCTTCCTGTCG-3′.

HEK293A cells were seeded on a 24-well plate and transiently transfected with the *C9ORF135* promoter reporter vector and SOX2 or OCT4 expression plasmids with Lipofectamine 2000. pRL-CMV (Promega) expressing Renilla luciferase was transfected as an internal control. After 48 h, the transfected HEK293A cells were lysed in passive lysis buffer, and luciferase activity was measured with the dual-luciferase reporter assay system (Promega) with a Centro LB960 96-well luminometer (Berthold Technologies, Baden-Württemberg, Germany).

### Mass spectrometry (MS) analysis

The collected hESCs were homogenized on ice in a lysis buffer supplemented with protease inhibitor cocktail^29^. The protein lysates were separated by SDS-PAGE. The gels were stained with 12 mM silver nitrate for 50 min and then washed in water 3 times for 20 min each. Selected bands were excised from the gel and analyzed by MS with an LTQ Orbitrap XL (Thermo Fisher Scientific, Waltham, MA, USA) according to the manufacturer’s instructions.

### Early stage of hESC differentiation via embryoid bodies (EBs)

Human ESCs H1 were cultivated in Essential 8 medium (Life Technology) on Matrigel (BD Biosciences)-coated plates^30^. To induce spontaneous differentiation, the H1 cells were digested with Accutase into single cells, then plated onto low-adhesion 6-well plates supplemented with Rock inhibitor, 10 μM Y27632 (Tocris). After 24 h, EBs were formed and cultured in E6 medium (Life Technology) without bFGF and TGFβ. To induce designated differentiation toward the three germ layers, the EBs were subjected to the designated induction conditions. For ectoderm differentiation, the EBs were maintained in neurobasal medium supplemented with 1% N2, 1% B27, 1% NEAA, 2 mM glutamine, 100 U/mL penicillin, 100 mg/mL streptomycin (all reagents from Life Technology), and cocktail of factors of 10 μM forskolin, 1 μM Compound C and 3 μM CHIR99021 (all from Peprotech) according to previously published protocols with some modifications^31^,^32^. For mesoderm differentiation, the EBs were cultured in RPMI1640 medium supplemented with 1% B27 (without Vitamin A) and 100 ng/mL BMP4, as described by Yung’s protocol with some modifications^6^. For endoderm differentiation, the EBs were replaced with DF12 medium supplemented with 0.1% BSA and a cocktail of 100 ng/mL Activin, 25 ng/mL Wnt 3a and 50 nM PI103 (all from Peprotech) only on the first day. The cells were subjected to subsequent treatments with only 100 ng/mL Activin for another 3 days as described by Melton’s protocol with some modifications^33^. After 4 days of directed differentiation, EBs were digested into single cells with Accutase (Life Technology), and the resultant cells were plated on the Matrigel-coated glass bottom dishes. Immunofluorescence assays were performed with lineage commitment markers. The cells were observed under a confocal microscope (LEICA STP6000, Germany).

## Additional Information

**How to cite this article:** Zhou, S. *et al. C9ORF135* encodes a membrane protein whose expression is related to pluripotency in human embryonic stem cells. *Sci. Rep.* **7**, 45311; doi: 10.1038/srep45311 (2017).

**Publisher's note:** Springer Nature remains neutral with regard to jurisdictional claims in published maps and institutional affiliations.

## Supplementary Material

Supplementary Information

## Figures and Tables

**Table 1:** Overlapping top probesets or genes in hESCs.

Probesets	Gene ID	Overlapping number of probesets	Overlapping number of genes	Gene symbol	Gene names
243610_at	138255	7	7	C9orf135	chromosome 9 open reading frame 135
237911_at		6	6	BF057809	Transcribed locus
220184_at	79923	6	6	NANOG	Nanog homeobox
239975_at	3116	5	5	HLA-DPB2	major histocompatibility complex class II DP beta 2
216379_x_at	100133941	6	6	CD24	CD24 molecule
225846_at	54845	5	6	ESRP1	epithelial splicing regulatory protein 1
237192_at		5	5	AI435590	Transcribed locus
231381_at	790952	5	5	ESRG	embryonic stem cell related (non-protein coding)
230195_at	100131138	5	5	LINC01405	long intergenic non-protein coding RNA 1405
206286_s_at	6998	5	5	TDGF1	teratocarcinoma-derived growth factor 1
203453_at	6337	5	5	SCNN1A	sodium channel of non-voltage-gated 1 alpha subunit
1559280_a_at		5	5	AA483467	CDNA FLJ35259 fis clone PROST2004251
219955_at	54596	4	5	L1TD1	LINE-1 type transposase domain containing 1
1553874_a_at	84891	4	5	ZSCAN10	zinc finger and SCAN domain containing 10
231061_at		4	4	AI671581	Transcribed locus
230597_at	84889	4	4	SLC7A3	solute carrier family 7 member 3
214974_x_at	6374	4	4	CXCL5	chemokine (C-X-C motif) ligand 5
214240_at	51083	4	4	GAL	Galanin GMAP prepropeptide
210265_x_at	642559	4	4	POU5F1P3	POU class 5 homeobox 1 pseudogene 3
206424_at	1592	4	4	CYP26A1	cytochrome P450 family 26 subfamily A polypeptide 1
206309_at	11061	4	4	LECT1	leukocyte cell derived chemotaxin 1
206268_at	10637	4	4	LEFTY1	left-right determination factor 1
206002_at	10149	4	4	ADGRG2	adhesion G protein-coupled receptor G2
204891_s_at	3932	4	4	LCK	LCK proto-oncogene Src family tyrosine kinase
1554777_at	132625	3	5	ZFP42	ZFP42 zinc finger protein
242128_at	5015	3	4	OTX2	orthodenticle homeobox 2
235199_at	54941	3	4	RNF125	ring finger protein 125 E3 ubiquitin protein ligase
228038_at	6657	3	4	SOX2	Sex determining region Y box 2
206653_at	10622	3	4	POLR3G	polymerase RNA III polypeptide G
204286_s_at	5366	3	4	PMAIP1	phorbol-12-myristate-13-acetate-induced protein 1
201131_s_at	999	3	4	CDH1	cadherin 1 type 1 or epithelial E-cadherin
229724_at	2562	2	4	GABRB3	gamma-aminobutyric acid A receptor b3
202310_s_at	1277	2	4	COL1A1	collagen type I alpha 1

Note: Some single genes are represented by several Affymetrix probesets. We combined the probesets for the same genes in the screening datasets.
