# Supplementary material for: C9ORF135 encodes a membrane protein whose expression is related to pluripotency in human embryonic stem cells
Source: Sci Rep. 2017 Mar 27;7:45311. doi: 10.1038/srep45311 (PMC5366912; doi:10.1038/srep45311)
Supplement: Supplementary Information [file srep45311-s1.pdf]

Supplementary Information

C9ORF135 encodes a membrane protein whose expression is related to pluripotency in human embryonic stem cells

Shixin Zhou[1], Yinan Liu[1], Yumin Ma[2], Xiaoyan Zhang[1], Yang Li[1], and Jinhua Wen[1,*]

1. Department of Cell Biology and Stem Cell Research Center, School of Basic Medical Sciences, Peking University Health Science Center, Beijing 100191, China
2. Department of Endocrinology and Metabolism, Peking University People's Hospital, Peking University Diabetes Center, Beijing 100044, China

Table S1, RT-qPCR primers used to examine hESC differentiation

Genes	Forward primers	Reverse primers
C9ORF135	5'TGGTGTATTCCTGGCACCGT3'	5'GGGATTCATCGGTTCCCAGT3'
Nanog	5' TGGCGCGGTCTTGGCTCACT 3'	5' AGGTGGCGGGCGCCTGTAG 3'
Nestin	5'CCTGGGAAAGGGAGAGTACC3'	5'TGGTCCTTCTCCACCGTATC3'
Pax6	5'TCCATCTTTGCTTGGGAAATC3'	5'TAGCCAGGTTGCGAAGAACT3'
Brachyury T	5'GCAAAAGCTTTCCTTGATGC3'	5'ATGAGGATTTGCAGGTGGAC3'
KDR	5'CGGCTCTTTCGCTTACTGTT3'	5' AGCATGGAAGAGGATTCTGG3'
FOXA2	5' GAGAAGAAATCCATAACACC3'	5' TTCTTTCCCGTTTTCCTCCT3'
SOX17	5'AGCAGAATCCAGACCTGCAC3'	5'TTGTAGTTGGGGTGGTCCTG3'

Figures and Figure Legends

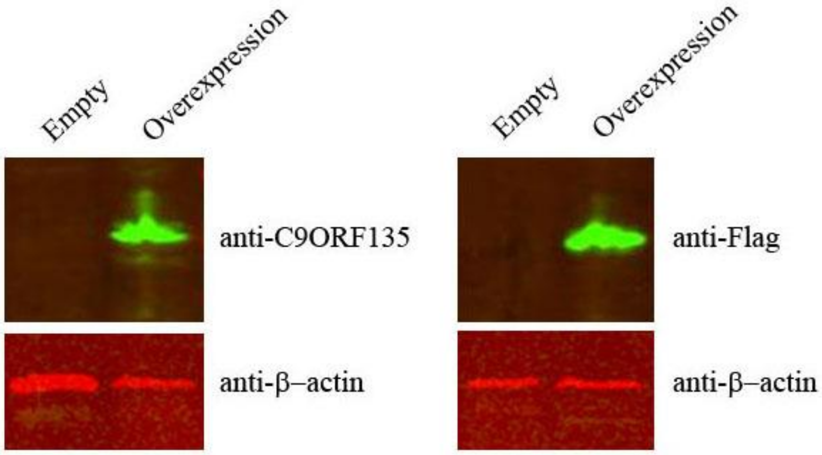

Figure S1. The antibody against C9ORF135 displayed specificity to the target protein. We expressed exogenous full-length *C9ORF135* with an N-terminal FLAG tag and the empty vector in HEK293A cells. The harvested cell lysates were analyzed via western blotting. The left panel shows detection with anti-C9ORF135 antibody, and the right panel shows detection with anti-Flag-antibody. β-Actin was used as a loading control.

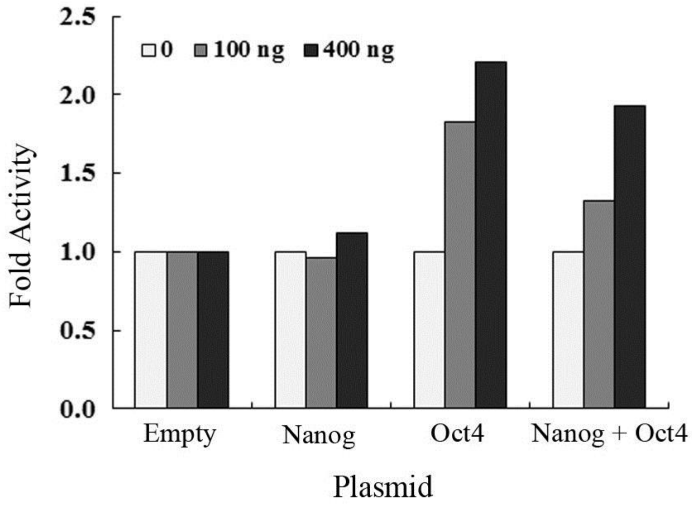

Figure S2 *C9ORF135* promoter activity in the presence of OCT4 and Nanog. A luciferase reporter containing the *C9ORF135* proximal promoter was used to monitor transcriptional activity in the presence or absence of OCT4 and Nanog vectors.

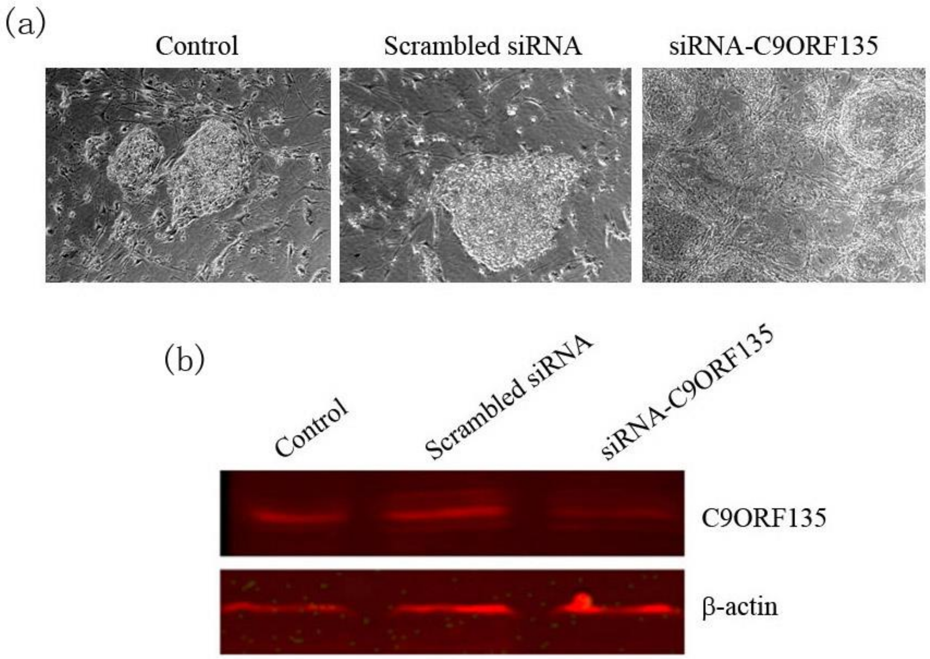

Figure S3. Morphology (a) and western blot analysis (b) of hESC clones after *C9ORF135* shRNA knockdown. The control shows the hESC protein level. The hESCs were transfected with lentivirus containing scrambled shRNA and shRNA against C9ORF135. The hESCs displayed morphological spreading after being subjected to *C9ORF135* shRNA knockdown (Western blot). β-actin was used as a loading control.
